# A Single-Case Design Investigation for Measuring the Efficacy of Gestalt Therapy to Treat Depression in Older Adults with Dementia in Italy and in Mexico: A Research Protocol

**DOI:** 10.3390/ijerph19063260

**Published:** 2022-03-10

**Authors:** Alessandra Merizzi, Rosanna Biasi, José Fernando Álvarez Zamudio, Margherita Spagnuolo Lobb, Mirko Di Rosa, Sara Santini

**Affiliations:** 1 Centre for Socio-Economic Research on Aging, IRCCS INRCA-National Institute of Health and Science on Aging, Via Santa Margherita 5, 60124 Ancona, Italy; s.santini2@inrca.it; 2 Istituto di Gestalt HCC Human Communication Centre Italy, Via S. Sebastiano 38, 96100 Siracusa, Italy; rosanna.biasi@gestalt.it (R.B.); margherita.spagnuolo@gestalt.it (M.S.L.); 3 Instituto Humanista de Psicoterapia Gestalt (IHPG), Africa 6, La Concepción, Mexico City 04020, Mexico; fernandoalvarezzamudio@gmail.com; 4 Unit of Geriatric Pharmacoepidemiology and Biostatistics, IRCCS INRCA-National Institute of Health and Science on Aging, Via Santa Margherita 5, 60124 Ancona, Italy; m.dirosa@inrca.it

**Keywords:** Gestalt therapy, dementia, depression, single-case experimental design, psychosocial interventions

## Abstract

Psychotherapy is one of the evidence-based clinical interventions for the treatment of depression in older adults with dementia. Randomised controlled trials are often the first methodological choice to gain evidence, yet they are not applicable to a wide range of humanistic psychotherapies. Amongst all, the efficacy of the Gestalt therapy (GT) is under-investigated. The purpose of this paper is to present a research protocol, aiming to assess the effects of a GT-based intervention on people with dementia (PWD) and indirect influence on their family carers. The study implements the single-case experimental design with time series analysis that will be carried out in Italy and Mexico. Six people in each country, who received a diagnosis of dementia and present depressive symptoms, will be recruited. Eight or more GT sessions will be provided, whose fidelity will be assessed by the GT fidelity scale. Quantitative outcome measures are foreseen for monitoring participants’ depression, anxiety, quality of life, loneliness, carers’ burden, and the caregiving dyad mutuality at baseline and follow-up. The advantages and limitations of the research design are considered. If GT will effectively result in the treatment of depression in PWD, it could enrich the range of evidence-based interventions provided by healthcare services.

## 1. Introduction

Dementia is a global epidemic; it is estimated that there are over 55 million people living with dementia worldwide, with the number expected to almost triple by 2050. Most of this increase can be attributed to population ageing, which is occurring at an unprecedented rate in low- and, particularly, middle-income countries [1].

Dementia, also called major neurocognitive disorder, is an umbrella term that describes a set of symptoms of cognitive, psychological, and behavioral nature that compromise the daily functioning of a person, the underlying cause of which may be one or more neurocognitive diseases, of which Alzheimer’s disease (AD) is the most common. Dementia can affect people at any age, but its prevalence increases significantly over time, particularly after the age of 65, doublings every five years [2].

There is a correlation between ageing, dementia, and depression. Depression predominantly affects older people, with the prevalence increasing to 9.3% in over 60 s [3]. Changes related to age and chronic physical diseases may contribute to modifications in the brain areas involved in the regulation of mood, i.e., the frontostriatal circuits, amygdala, and hippocampus, respectively, predisposing to depression [4]. Moreover, isolation, relocation, caregiving, and other age-related psychosocial stressors are a significant risk factor for depression in later life [5]. Chronic stress can increase cortisol levels, which contribute to depressive disorders and the atrophy of the hippocampus, also affecting its cognitive functions [6,7,8].

Literature shows that the association between depression and dementia is complex and bidirectional. Longitudinal studies suggest that recurrent depressive symptoms in adulthood, late-life depression, and chronic subsyndromal depression significantly increase the risk of dementia over time [9,10,11]. Therefore, early-life depression may be considered a risk factor for dementia in older age, and late-life depression may be a prodrome to dementia. Likewise, dementia can be considered a risk factor for depression. People with dementia (PWD) have a higher rate of overt depression, i.e., 20% in AD, 37% in frontotemportal dementia, and 50% in vascular dementia [12,13,14], whilst depressive symptoms have been reported in 30–50% of AD patients [15].

Depression in dementia can be underdiagnosed because its presentation in older people can differ from the Diagnostic and Statistical Manual of Mental Disorders (DSM)-5 [16] criteria, which are mostly based on adult populations, and the cognitive symptoms may overlap with the dysfunctional signs of dementia [17].

### 1.1. Dementia as a Social Disease: The Impact of Dementia on Informal Carers’ Health and Social Life

Dementia affects the person suffering from it, as well as all his/her family and social network. In fact, most PWD live at home assisted by their family members (e.g., spouses and children) who become their main carers [18].

Beyond the multiple cognitive impairment, the behavioural and psychological symptoms experienced by PWD, such as depression, personality changes, aggressiveness, delusions, hallucinations, wandering, and sleep disturbances [19,20], are challenging to accept and manage by informal carers. The latter, therefore, often tend to reduce the occasions on which they can be in public, in order to avoid embarrassing situations, thus leading them to social isolation, defined as the lack of social contact, relationships, and integration [21,22,23]. PWD are considered one of the most socially excluded groups of older people [24,25], and so are their informal carers [26], with a negative impact on their health-related quality of life [27]. The association between social isolation and dementia is bilateral because the former is both the consequence and one of the driving forces behind the latter. Empirical evidence greatly highlights the association between social isolation and dementia and vice versa [28]. Family carers of PWD subsequently report increased rates of caring strain, physical and mental health problems, and experience difficulties in maintaining employment, leisure activities, and family interactions, all of which has an impact on their quality of life [28]. Moreover, family carers can experience fatigue, frustration, a sense of inadequacy, feelings of being trapped, and loneliness, with the latter worsened by social exclusion and stigma characterising the care recipients. Furthermore, increased caregiver burden, in relation to behavioural or depressive symptoms in dementia, has been shown to lead to neglect or physical and psychological abuse by the family carer [29,30]. With the advent of the COVID-19 pandemic, older people, including PWD, have been called to shelter because they have been identified as being at high risk of contracting the SARS-CoV-2 infection [31], and the rules around social distancing have restricted access to community services for PWD and their carers. During the outbreak, few care services have adapted to providing remote support to PWD [32], and most centres for cognitive disorders and dementia markedly reduced their activities. As a consequence, many PWD experienced faster symptom deterioration during the pandemic [33]. Social isolation caused a decline in memory function, increased anxiety and depression symptoms (especially in individuals with mild-to-moderate dementia), and worsened the general health condition of informal carers who experienced increased tiredness, feelings of being overwhelmed, health problems, sleep disturbances, and greater irritability associated with social isolation [34]. A clear association has been found between the cognitive decline of older PWD during the lockdown and increase of family carers’ psychological distress [35].

In light of the above, psychotherapy should involve the person in need, as well as the family carer, as an individual belonging to his/her environment, in order to put in place appropriate measures for treating dementia and correlated depressive symptoms. GT prioritising the relationship between the person (and his/her social contacts) and environment may be an effective approach for mitigating the disruptive impact of depression on PWD and family carers, as described in the paragraphs below.

### 1.2. The Contest of Dementia Diagnosis and Formal Care: A Snapshot of Italy and Mexico

The care and the management of dementia differs country-by-country, depending on epidemiological figures, the long-term care (LCT) system, welfare regimes, and level of country income. In Latin America, the prevalence of diagnosed dementia in 2010 was 3.10 million cases, compared to 9.95 million in Europe, and an increase of 393% is expected by 2050, compared to an 87% rise in Europe [36]. The increasing number of diagnosed PWD will contribute to growing inequalities in low- and middle-income countries, where 15% of dementia-related costs is covered by social care, compared to 40% in high-income countries [37].

Despite Europe being considered a wealthy continent with a better health system, compared to Latin America, the quality and level of care provided to PWD, from the time of diagnosis onwards, can differ significantly in each country.

This article shows an intervention research protocol, targeted to PWD and depressive symptoms living in Mexico and in Italy. Since these countries have different LTC systems and gross domestic products (GDP), the epidemiological and socio-economic contexts have been explored and are described in the following sub-paragraphs, in order to provide the rationale of the study protocol presented in this paper.

In Italy, the overall number of PWD will almost double from 2018 to 2050, reaching the figure of 2,247,715 individuals, which will represent 4.13% of the overall population, compared to 2.12% in 2018 [38,39], thus keeping in line with the European and western world trend. In the country, diagnosis of dementia is frequently made in hospital, when the person is at a middle-advanced stage of the illness, contrary to what happens in other European countries, such as the United Kingdom, where health services promote early diagnosis, and memory services have a great focus on providing support at the early stages of the disease. Although the intent is to slow down the progress of the disease, in Italy, there is neither a clear strategy to facilitate early diagnosis nor to promote a person-centred care aimed at keeping the person independent and cutting the cost of LTC. The diagnosis of dementia is often not even communicated to the patients, but only to their family members, and working on acceptance of the new condition can be problematic.

Once the diagnosis is received, families often attempt to keep the PWD at home and manage the care assistance by using support from both public and private sources. The National Health Service offers home healthcare services (Assistenza Domiciliare Integrata, ADI), which include specialist assistance from nurses, physiotherapists, and medical doctors; yet this is only for an average of 16 h per year, per patient, and does not include psychotherapy [40]. The last available data reported that only 9.5% of PWD received this type of support in 2016 [41]. At a local level, the municipality partially offers, through a voucher scheme, another type of home social care service (Servizio di Assistenza Domiciliare, SAD) that covers basic individual needs, e.g., food intake for highly dependent older people. The provision of SAD can vary significantly across Italian areas and is not well-integrated with the ADI [42]. Moreover, it is neither considered a sufficient nor appropriate service for covering the needs of PWD and their family carers [43]. Another type of dementia care voucher, which is not distributed evenly across the country, consists of funding to access a psycho-educational intervention in “Alzheimer Day Centers”, available only to 12.5% of families, despite this being part of an essential level of assistance [41]. Finally, the state care allowance (Indennità di Accompagnamento) is a universal and not means-tested monetary transfer of 522.10 EUR per month, provided to the person in need of care. Such a contribution often partially covers the cost of live-in (migrant) care workers who can guarantee H24 home assistance to PWD [41]. This means that families cover most of the cost of care with their own budget. [44].

Epidemiological figures in Mexico mirror Latin America’s trend, with a prediction of nearly 1.5 million cases by 2030 and increase of 414% between 2010 and 2050 [45]. Only in recent years has there been a governmental effort to boost the proposal of the first national Alzheimer’s Plan of Action (APA) [46]. The APA aims at promoting PWD and their families’ well-being by empowering services of the Mexican healthcare system and cooperating institutions. Guidelines and strategies for implementing specific actions have been published, the second strategy being “To ensure access to quality services” [46,47]. However, currently, many families in rural areas and living in poverty neither have access to specialised dementia centres nor training and guidance for home care assistance. Community-type care has emerged as a response and alternative. However, the aim of this type of support is limited to providing information to sensitise the community, and lacks an ethical approach, based on empirical research, prior to applying the intervention.

### 1.3. Psychological Support and Psychotherapy Interventions: Gestalt Therapy View on Dementia and on Depression

In light of the above, very little is offered, in terms of psychological support, for PWD and their families to hinder depression, both in Italy and in Mexico, as well as when PWD live at home and in residential or semi-residential institutions. Conversely, the treatment of depression is very important because depression affects the relationship between PWD and their family, as the person becomes more apathetic and less cooperative. As a result, it becomes even more difficult for family members to take care of the person with dementia, with negative effects on the health of the informal carer (and level of care required), social dimensions (i.e., social inclusion and participation), and, ultimately, progression of the disease. In fact, some studies have underlined the association of depression in dementia with decreased quality of life [48] and level of autonomy in performing the instrumental activities of daily living (IADL) [49], quicker progression of cognitive decline, earlier admission to residential facilities [50], and, as described above, a clear association to the interruption of social relationships [27]. In light of this, it is crucial to identify the symptoms of depression early in people with dementia and offer them appropriate psychotherapeutic support.

Gestalt therapy (GT) is a phenomenological, aesthetic, and field-oriented approach based on humanistic and holistic principles. GT theory considers that, through human development, individuals learn ways of coping and relating to others in their family environment, the learning of which becomes then generalised and automatic, inscribed in the procedural memory that tends to be economically fast for our brain and kept out of awareness [51]. The primary aim of GT is to restore the wholeness of the experience of the person, including bodily feelings, movements, emotions, and the ability to creatively adjust to environmental conditions by empowering the awareness in the present moment. The therapist works to address the implicit knowledge of patients by accessing her/his non-verbal and creative resilience. The *phenomenological* principle addresses the experience of the client in its wholeness by paying attention to nonverbal cues over the cognitive ones. For instance, rather than focusing on the memory of an event, the emphasis is on developing the ability to stay in the therapeutic relationship harmonically, like in a dance, and to learn how to express their own wish for relational contact and sense of agency. The *aesthetic* principle allows therapists to look at the client’s experience through all human senses; instead of using cognitive criteria to evaluate the capacity for relational contact of the client, the therapist can consider other resilient aspects, such as their body movements, emotions, level of arousal, and direction of energy (intentionality for contact) [52]. The *field* principle allows the therapist to work with reciprocity and consider synchronicity as a cue to evaluate the ability of the client [53]. An effective intervention has to be focused on the relational “dance” that therapist and client co-create, more than on achieving a certain individual goal. Whilst a goal is agreed upon at the time of the therapy contract, the focus of the work will not be on the goal, which will only be discussed at review points and the end of therapy. The “dance of reciprocity” will also enable the client to fulfil cognitive goals because this relational “dance” will support their ability to cope and adjust to the environment [52]. In this way, the intervention will be on the function of the client to flexibly adapt to their environment, in different and creative ways, rather than on acquiring certain behavioural aims.

Because of Gestalt therapy’s experiential and experimental nature, there is a lack of empirical research supporting its evidence. A recent systematic review selected eleven scientific papers on the effectiveness of the approach, confirming the validity of GT as a clinical model [54]. In favour of the use of GT with PWD, it is possible to consider some neuropsychological and relational aspects of the approach, together with the common residual abilities of PWD. First of all, it is important to take into account that, although working memory, short-term memory, and language can become impaired with the progression of dementia, emotional and procedural memory are retained skills that may decay only at the very late stages of the disease [55]. GT focuses on procedural memory, or nondeclarative memory, which is related to embodied learning on how things are done, through the selection, use, retention, and recall of perceptual, cognitive, and motor skills [51]. This procedural level should be the priority in the therapeutic process when working with PWD and, in general, to promote a long-term change. Gestalt therapists work on attunement to movement, facial expressions, posture, tone of voice, the affective content of communication, and supporting difficult emotions that emerge in the relational field with the patient [56]. By focusing on the importance of the person’s adaptation to the new situation (neurological, emotional, and social), Gestalt therapists help the patient to create new responses to the changing environment, thus addressing the social dimension of the illness [54] by supporting resources, instead of being concerned with what does not work and continuous losses. The changing environment for PWD relates not only to the outside world, but also to their experience of self, in relation to the change and challenges that dementia brings, which adds a level of complexity when working with these patients.

Considering the aims of this study, it is also relevant to define depression in GT, which is viewed from a phenomenological and aesthetic perspective [57]. GT offers an original attempt to reformulate depression, in terms of the experience of “contact” with the environment. “Contact” in GT is the ability to interact with the environment with the aim to fulfil a need. GT connects depression with five dimensions, related to the organism/environment field, which have also been observed to co-occur in both the client and the therapist: (1) depression is considered the lack of connection with the environment; (2) a depressive experience is viewed as the renunciation of the desire to be desired, rather than related to the frustration of past desires not being fulfilled; (3) depression is seen as the inability to transcend oneself; (4) depression is conceptualised as a lack of hope, rather than a lack of happiness; (5) depression indicates the inability to feel oneself—from the bodily sensations level to the emotional one [58].

The effectiveness of GT for depression has been supported by a small number of studies, one of which involved geriatric patients with depression and anxiety [54,59]. In fact, despite the potential effectiveness of this approach, there is still a gap in research on using GT for PWD and those with depressive symptoms.

The experiential, creative, and flexible aspects of GT can provide a unique way of working therapeutically with PWD because of the focus on the non-verbal part of the experience, residual abilities of the person, and use of body movement and art as aims of communication, which can greatly facilitate the therapeutic engagement towards the attainment of therapeutic goals.

GT can offer a way of working with PWD and depression that can easily incorporate access to other forms of support and activities, not only in a talkative way, but in a practical one. The creative flexibility implicit in the approach allows therapist and patient to develop the therapeutic path together, which can be extended to the outside world, therefore going beyond the walls of the therapy room. For instance, they can agree that, as part of therapy, they attend a walking group or go for a bus trip together.

### 1.4. Selection of the Research Design: Single-Case Experimental Design

Scientific literature offers plenty of evidence that non-pharmacological interventions are the best choice for managing the psychological and behavioural symptoms of dementia, including depression [60,61]. Pharmacotherapy for depression in dementia has unclear outcomes, and antidepressants may accelerate the progression of dementia [62,63,64]. Psychological therapies, particularly cognitive behavioural therapy (CBT), are among the most evidenced approaches to treat depression in dementia [65].

In a systematic review, Cheston and Ivanecka [66] highlight that there is poor evidence of the efficacy of psychotherapy for the treatment of non-cognitive symptoms in dementia patients. The authors state that manualised interventions, such as CBT, are best evidenced because the treatment can be measured through the RCT model, yet RCTs have important limitations in psychotherapy research. The American Psychological Association [67] states that the single-case time series design can be as valid as RCTs. Moreover, single-case experimental designs (SCEDs) can ensure high-quality research for small heterogeneous groups in clinical settings, allowing for the focus on the unique needs of patients [68], rather than on a set of given techniques. It is well-known that dementia affects each person differently; therefore, it is crucial to adopt person-centred approaches in dementia care and tailor the psychotherapeutic treatment to the needs and presentation of each patient. Gestalt psychotherapy can be a flexible and user-centred approach that, for the reasons mentioned above, could be proved efficient in treating depression in dementia. Considering the qualities of GT introduced above, SCEDs appear to be the best type of investigation to measure the effectiveness of the Gestalt approach.

### 1.5. Proposed Research Aims and Objectives

The general objective of the research is to determine whether or not GT is an effective treatment for depression in PWD.

Specific objectives of the study are:

- To assess if there is a pre/post improvement and, if so, how great this is (clinical significance), according to the outcome variables rates, as well as whether it can be attributed to the therapy intervention.
- To assess if study participants suffer with loneliness.
- To assess if loneliness is mitigated by the regular contact with the therapist.
- To assess if the regular contact with the therapist affects the level of depression.
- To assess if a clinical improvement of depression symptoms has an indirect effect on the level of family carers’ burden.
- To assess if a clinical improvement has an indirect effect on the mutuality of the relationship between the patient and the family carer.

In order to achieve the objectives, the following research questions will need to be answered:

1. Is there any pre/post treatment improvement?
2. If the answer to question 1 is yes, is the change clinically significant (reduction of symptoms by 50%)?
3. If loneliness is a factor, is the improvement solely due to the social contact with the therapist? OR
4. Can the improvement be attributed to the therapy intervention?
5. Can the improvement of the patient also have an impact on the caregiver’s burden and level of mutuality?

The researchers hypothesised that: depressive symptoms will improve post GT treatment and the improvement will be maintained at follow-up; the level of loneliness, if a factor, can decrease post-treatment and remain so at follow-up; the improvement of symptoms is related to the GT treatment; the level of burden of the family carer will reduce whilst the mutuality in the relationship will increase as the PWD’s depression improves.

## 2. Materials and Methods

### 2.1. Research Design

The ABA single-case experimental design will be used for the purpose of the study, as explained previously.

**Figure 1:**
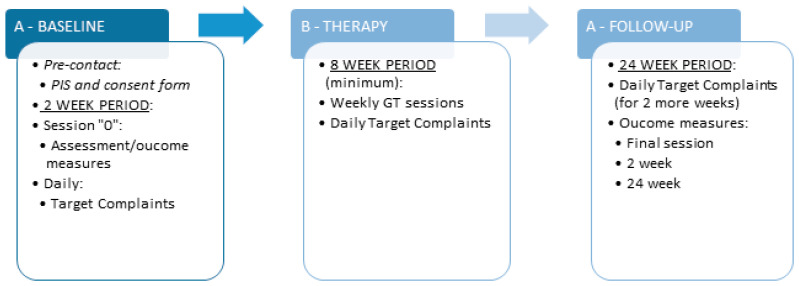
ABA design of the study.

The research method is divided into three phases: an “A” baseline period of two weeks between the assessment session “0” and initial psychotherapy session; a “B” intervention period of minimum eight GT sessions; an “A” follow-up period of six months after the end of the intervention, with measurements at the final session, after two weeks and six months. More specifically, in relation to data collection, the following will happen in each phase: A—at the baseline, outcome measures will be administered at session “0”, and individual target complaints (TC) will be recorded daily for the following two weeks; B—in the intervention phase, target complaints will be completed daily; A—in the follow-up phase, participants will continue with daily assessment for another two weeks, while outcome measures will be administered at the final session, i.e., week two and week twenty-four (Figure 1).

### 2.2. Study Setting and Intervention

The intervention will consist of individual GT sessions, delivered weekly for the duration of one hour each. The minimum number of sessions is eight, whilst a maximum number will be recorded, depending on the individual needs of the participants with dementia.

The intervention will be delivered in psychotherapeutic settings, provided by public health clinics or private therapy centres.

Participants will be provided with an information sheet describing the study purpose and procedure, and they will be asked to sign a consent form for data collection and treatment. The full anonymity of the individuals taking part in the treatment will be ensured, according to the general data protection regulation (2016/679) [69] and national laws on the privacy of sensitive and health-related data, currently in force in Italy and in Mexico. The study protocol will be submitted to the attention of the competent ethics committees.

### 2.3. Sample and Recruitment

Recruitment will involve two groups of participants: 6 Gestalt psychotherapists and 12 PWD suffering from depression, with 6 in Italy and 6 in Mexico, respectively. The sample size has been chosen on the basis of recommendations provided for SCED in GT, which state that a number of 4 to 10 cases can give the study an international validation for the efficacy of the method for a specific type of problem [70]. For this research, the study number is slightly higher to include a dropout rate of 20–30%. In line with the above, the researchers decided to have as an inclusive approach as possible, with the twofold aim of giving a chance of treatment to patients with different types of dementia (i.e., for compassionate reasons) and observing its impact on them (i.e., for research interest).

To be part of the study, PWD will need to: (a) provide evidence of a formal diagnosis by a medical doctor, i.e., a neurologist, geriatrist, or psychiatrist; (b) have a clinical dementia rating–sum of the boxes (CDR-SOB) score of 3–4 (very mild dementia) or 4.5–9 (mild dementia) [71]; (c) be able to engage in therapy; (d) display symptoms of mild depression, i.e., geriatric depression scale (GDS) score 10–19 [72] and/or anxiety (neuropsychiatric inventory anxiety subscale, NPI-A score ≥ 4 [73]); (e) maintain the psychopharmacological treatment during the investigation; f) live in the community.

Exclusion criteria are: (a) diagnosis/symptoms of dementia at a moderate–severe stage (given by a medical specialist) or CDR-SOB score of 9.5–18; (b) display of challenging behaviours, delusions, or psychotic symptoms or major depression (GDS score between 20–30); (c) high level of risk and safeguarding issues; (d) parallel therapy/intervention for the same target complaint (e.g., psychoeducational groups); (e) variation in psychopharmacological treatment during the investigation; (f) admission to hospital/residential care.

Gestalt psychotherapists included will need to: (a) agree to participate, be recorded during sessions, and fill in questionnaires; (b) be state licensed Gestalt psychotherapists, with previous experience of working with PWD; (c) have access to supervision with a Gestalt supervisor.

PWD will be recruited through public health clinics in Italy and Mexico. The research team will contact health centres with a request to inform eligible patients (according to the judgement of their specialist doctor) about the study; possible participants can then contact the team themselves or agree to have their contact information forwarded to the researcher. Recruitment will be carried out by Gestalt psychotherapists, who are also clinical psychologists with experience in the field of neuropsychology and dementia. Gestalt psychotherapists will be recruited through national professional bodies and organizations.

### 2.4. Measures and Data Collection Tools

Overall, data will be collected through questionnaires and audio/video recording. Outcome measures will gather quantitative data on the symptoms, quality of life, level of loneliness, family carers’ burden, and mutuality and will be administered at session “0”, at the final session and follow-up (week 2 and week 24), whilst target complaints will be repeated daily and used for the time series analysis. All the measures and related administration time points are shown in Table 1 and described below.

The Neuropsychiatric Inventory Questionnaire (NPI) was developed by Cummings et al. [73] and validated by Binetti et al. [74] for the Italian population and by Zepeda et al. [75] for the Mexican population. It measures the behavioural symptoms related to dementia conditions, assessing twelve sub-domains: delusions, hallucinations, agitation/aggression, depression/dysphoria, anxiety, elation/euphoria, apathy/indifference, disinhibition, irritability/lability, motor disturbance, night-time behaviours, and appetite/eating. Additionally, a caregiver distress scale was included for measuring the psychological impact of the neuropsychiatric symptoms on family caregivers [76]. The NPI anxiety (NPI-A) score will be used to assess the level of anxiety of participants at pre/post treatment. The test has good internal consistency, content validity, and inter-rater/test-retest reliability.

The clinical dementia rating (CDR) is a multidimensional tool that measures the level of cognitive and functional impairment from the patient and proxy point of view. The information is collected by independent semi-structured interviews. Assessed domains include: memory, orientation, judgment and problem solving, community affairs, home and hobbies, and personal care. The score ranges from no impairment to severe dementia on a five-point scale. For the purpose of this project, the “sum of the boxes” (SOB) scoring method will be used: each domain box scores are summed, giving the possibility to differentiate between very mild (3–4) and mild dementia (4.5–9). CDR is widely used in research for its well-established validity and reliability [77,78]; additionally, the CDR-SOB method gained good reliability [71,79] and reputable use of its indexes of severity in therapeutic trials [80]. In this research, CDR will be used to assess the level of dementia at the recruitment phase, then to observe any natural progression of dementia, as a confounded variable at follow-up.

The geriatric depression scale (GDS) is a screening tool consisting of a 30-item questionnaire for measuring symptoms of depression in older people. Its cut-off score is 9/30 and mild depression ranges between 10–19, whilst severe depression between 20–30. It is widely used in research and clinical settings because of its sensitivity (92%) and specificity (89%). GDS seems reliable to detect depression in mild-to-moderate dementia, depending on the level of insight of the person; therefore, it needs caution in administration [72].

The target complaints (TC) daily form [81] is a co-constructed self-report measure of three central idiosyncratic issues and one general wellbeing item. The items are identified together with the therapist at session “0”, then rated daily on a Likert-scale, from 1 to 10, where 1 equals “not bothered at all” and 10 equals “could not be worse”. Each complaint should be concrete, measurable/quantifiable, frequent, stable without treatment, relatively independent from one another, specific, and negatively formulated. The main instruction that helps identifying and scoring the target complaints is “Please record up to three presenting concerns that brought you to therapy. Circle the number that best represents your personal degree of discomfort as a result of each issue.” Improvement on global assessments and target complaints correlated at 0.71 [82], and test-retest reliability was at 0.76 [83].

The University of California, Los Angeles loneliness scale version 3 (UCLA-LS3) is a 20-item, self-reported questionnaire, broadly used to measure subjective feelings of loneliness and social isolation in various populations, including older adults and people with dementia. UCLA-LS3 has a well-established validity and reliability for its use in different languages, among which are Italian and Spanish [84,85,86]. The scale will be used to assess whether loneliness is a present factor and to observe whether regular contact with the therapist, rather than treatment, can influence this secondary variable and, thus, the level of depression (primary variable).

The quality of life-Alzheimer’s disease (QOL-AD) is a self-report tool for the PWD and their proxy/caregiver, based on 13 items of multiple domains, including physical health, mood, marriage, and fun. QOL-AD has excellent internal consistency and inter-rater reliability, as well as good criterion and construct validity [87].

The Ad-hoc common assessment tool is a questionnaire aimed at obtaining information on the level of care required by the PWD. It is an idiosyncratic and non-standardised tool, containing questions such as the weekly hours of caregiving and type of formal or informal support involved.

Zarit burden inventory (ZBI) measures the level of caregiver burden and is widely used in dementia research for its good validity and internal consistency reliability (Cronbach’s alpha coefficient of 0.92) [88,89]. The revised version contains 22 items, each one endorsed on a five-point scale, ranging from 0 (never) to 4 (nearly always). It provides a total score; recently, the author identified subdomains and related scores using a concept mapping method. Subdomains are: burden in the relationship, emotional well-being, social and family life, finances, loss of control over one’s life (scaling and scoring version 6.0 [90]).

The mutuality scale [91] is a self-report questionnaire that measures the construct of mutuality, as defined by the authors: “the positive quality of the relationship between caregiver and care-receiver” (p. 376). The scale consists of 15 items, divided in subdomains (love, shared pleasurable activities, shared values, and reciprocity), rated on a five-point scale, ranging from 0 (not at all) to 4 (a great deal). High scores indicate high mutuality, which is associated with decreased caregiver’s stress [91,92,93] and lower caregiver burden [94].

Clinical outcomes in routine evaluation–outcome measure (CORE–OM) is a 34-item self-report measure of psychological distress, comprising of four domains: wellbeing, symptoms, functioning, and risk [95]. CORE–OM is widely used in psychotherapy research and clinical settings for its good reliability and test-retest stability [96]. A study on the older adult population reported that the tool can be used with people with mild cognitive difficulties and recommended the use of cut-off points, identified by the latter investigation, due to the risk of false positives [97].

The Gestalt therapy fidelity scale (GTFS) [98] is a 25-item tool assessing the treatment fidelity on a 25-min videotape of GT sessions. Video recordings are randomly selected at the end of the first treatment, and external assessors evaluate whether the treatment is Gestalt or not by scoring each item using a “yes” or “no” rate. In the case that an intervention falls under the cut-off score of 11, the therapist will be excluded from the research, and the intervention will be considered null for the purposes of this study.

### 2.5. Data Analysis

Continuous variables will be analysed and reported as mean ± SD (standard deviation) or median and IQR (interquartile range), on the basis of their distribution (assessed through Shapiro–Wilk test). Discrete variables will be reported as absolute frequencies and percentage.

In order to evaluate any pre/post improvements (from baseline to follow-up: at the final session, after two weeks and twenty-four weeks following the end of the treatment) in the main outcomes, i.e., depression and anxiety symptoms (GDS; NPI-A), quality of life (QOL-AD), and the therapy outcome (CORE–OM), three types of analysis will be carried out: (a) visual analysis, comparing the target complaint (TC) scores during the three phases; (b) mean difference from baseline to follow-up tested, by means of paired t-test or Mann–Whitney U test, according to the distribution of the outcome variable [99]; (c) mean baseline reduction (MBLR) for obtaining the effect size for single-case designs [100,101,102], as follows:

MBLR = (Mean of Baseline phase − Mean of Follow-up phase) × 100/Mean of Baseline phase.(1)

The same procedure will be applied for the secondary outcome, i.e., loneliness. Additionally, data collected from family caregivers (i.e., QOL-AD family version, caregiver burden scale, and the mutuality scale) during the three phases of the study will be compared with subjects’ outcomes, in order to evaluate indirect effects of the treatment, by means of Pearson or Spearman correlation coefficients, as appropriate.

Blind raters will assess the fidelity of the approach by rating a small sample of sessions through the GTFS.

A two-tailed *p* value < 0.05 will be considered significant. Data will be analysed using SPSS for Windows V24.0 (SPSS Inc., Chicago, IL, USA) and Simulation Modeling Analysis (SMA) for Windows V07.30.20 (ClinicalResearcher, Jeff Borckardt, Medical University of South Carolina).

## 3. Discussion

Depression in dementia can be treated with the use of psychosocial interventions, psychotherapy being part of this category. Any therapeutic approach that is used to work with PWD should be flexible, person-centred, and creative, in order to meet the needs of the person, as it has been well-underlined by Kitwood and followers [103,104]. These qualities are already central in GT and testify to the suitability of the approach for working with this population.

The design of this study mainly followed indications for the development of a SCED in GT, as recommended by GT authors with experience in research [70,105]. The aim of observing the effectiveness of the approach has been enriched by considering the context of the experience of living with dementia, which inevitably includes informal carers, who are often family members. Therefore, the benefits of receiving GT as treatment should also be observed from the perspective of the main family carer. This added aspect is in line with the GT field principle, i.e., that the embodied learning from the therapeutic “dance” can elicit relational adaptations (a *new dance*) in the family system.

Providing psychotherapeutic support from the earliest stages of dementia allows the patients to cope with the disease from the very beginning, integrating it into their lives and becoming protagonists of the future therapeutic choices. A grieving reaction, accompanied by an emotional crisis, which may include depression, may be experienced from the moment of diagnosis [106], as a response to it, or even before diagnosis [107], as the person’s life begins to be affected by the initial symptoms of dementia. The support received through a psychotherapeutic intervention can help the person to cope with losses, in order to create continuous new adaptations. GT’s holistic approach makes it possible to undertake a pathway to support the physiological, emotional, and cognitive ground of the patient that may be disrupted by the dementia condition.

It is expected that the results obtained from this research can submit significant data to support the impact between pre/post improvement, as well as whether the observed changes are clinically significant. It will be possible to review the attribution of these changes to the Gestalt therapeutic work.

An indirect effect on the family carers may be observed, as a result of the researched intervention, which may manifest in a reduction of their stress levels or emotional burden and, consequently, the improvement of the relationship with those they care for.

The statistical comparison strengthens the present evidence-based study on the efficacy of GT with people in these circumstances and opens a whole field of work and possibilities for undertaking future studies.

## 4. Limitations and Challenges of the Research Protocol

The project was developed prior to the start of the COVID-19 pandemic and only considered an in-person setting for face-to-face therapy. Since we do not have sight of when the pandemic will terminate and considering that this time could come in several years, the inclusion of only in-person settings can be an important limitation. On the other side, an online setting may bring restrictions to some valuable aspects of GT, particularly when applied to PWD, if we consider the cognitive difficulties of patients that already imply some limits. Additionally, people who live in Italy and Mexico tend to receive a diagnosis at the mid, if not late, stages of the condition, which means that they will experience further limitations in using and engaging in tele-therapy.

In relation to data collection, qualitative data may add value to quantitative figures. At this stage, the protocol only includes a quantitative methodology can guarantee rigor and reproducibility. As the therapy sessions will be video recorded, there might be a possibility of further analysis of the content of the meetings (with the consent of the participants), as part of a secondary research project.

The application of the protocol might encounter some challenges. First of all, there are not many services for PWD that offer psychotherapy; therefore, there is not a large number of professionals with experience in the field of dementia. Additionally, the Gestalt psychotherapy community is not as large as that of other approaches, such as psychoanalysis and cognitive-behavioural therapies, so it may be even more difficult to find Gestalt psychotherapists with expertise in dementia. Another challenge that may have an impact on recruitment may be the stigma of having psychological problems (particularly present in the current cohort of older people), as this adds to the stigmatic images related to the dementia condition; together, these challenges may hinder acceptance of the need for help. As a final point, the lack of public healthcare provision for PWD means that there is a small availability of free-of-charge psychotherapy treatments in both countries. Therefore, the application of this protocol will depend on the obtainment of a grant and/or the in-kind work of the professionals involved.

## 5. Conclusions

The objective of the study is to observe the potential of GT as an effective intervention for working with PWD and depressive symptoms. The scientific soundness of the SCED methodology will rigorously produce reliable results that, we believe, will confirm the validity of GT with the considered population. Detailed reporting of the protocol can support the replicability of the research in other countries and settings.

## Figures and Tables

**Table 1:** Outcome measures with related variables, subjects involved, and time of data collection.

Instrument	Variable	Point of View	When It Is Used
Neuropsychiatric inventory (NPI)	Neuropsychiatric symptoms (patient) and caregiver distress	Expert judge interviews caregiver	Session “0”
Clinical dementia rating (CDR)	Cognitive symptoms and dementia diagnosis	Expert judge interviews patient	Session “0” and follow-up
Geriatric depression scale (GDS)	Depressive symptoms	Patient self-report	Session “0”, final session and follow up
Anxiety indicated by an NPI-A score of 4 or more	Anxiety symptoms	Expert judge interviews Caregiver	Session “0”, final session and follow up
Target complaints (TC)	Specific results of therapy	Patient self-report (after co-construction of instrument with a therapist)	Co-constructed at session “0” Client completes questionnaire every day after that until follow up session
UCLA-LS3	Loneliness	Patient self-report	Session “0”, final session and follow up
QOL-AD	Quality of life	Expert judge interviews patient and family caregiver self-report	Session “0”, final session and follow up
Ad-hoc common assessment tool	Level of care	Family member self-report	Session “0”
Zarit burden inventory	Caregiver level of stress	Family member self-report	Session “0”, final session and follow up
The mutuality scale	Mutuality of relationship	Patient and caregiver self-report	Session “0”, final session and follow up
CORE–OM	Overall results of therapy	Patient self-report	Session “0”, final session and follow up
Gestalt therapy fidelity scale (GTFS)	Treatment fidelity	Expert judge observes therapy sessions	End of first therapy treatment

## References

[1] World Health Organization (2021). Global Status Report on the Public Health Response to Dementia.

[2] Hugo J., Ganguli M. (2014). Dementia and cognitive impairment: Epidemiology, diagnosis, and treatment. Clin. Geriatr Med..

[3] Sjöberg L., Karlsson B., Atti A.R., Skoog I., Fratiglioni L., Wang H.X. (2017). Prevalence of depression: Comparisons of different depression definitions in population-based samples of older adults. J. Affect. Disord..

[4] Sexton C.E., Mackay C.E., Ebmeier K.P. (2013). A Systematic Review and Meta-Analysis of Magnetic Resonance Imaging Studies in Late-Life Depression. Am. J. Geriatr. Psychiatry.

[5] Alexopoulos G.S. (2005). Depression in the elderly. Lancet.

[6] Butters M.A., Young J.B., Lopez O., Aizenstein H.J., Mulsant B.H., Reynolds C.F., DeKosky S.T., Becker J.T. (2008). Pathways linking late-life depression to persistent cognitive impairment and dementia. Dialogues Clin. Neurosci..

[7] Sierksma A.S., van den Hove D.L., Steinbusch H.W., Prickaerts J. (2010). Major depression, cognitive dysfunction and Alzheimer’s disease: Is there a link?. Eur. J. Pharmacol..

[8] Wolkowitz O.M., Epel E.S., Reus V.I., Mellon S.H. (2010). Depression gets old fast: Do stress and depression accelerate cell aging?. Depress Anxiety.

[9] Dotson V.M., Beydoun M.A., Zonderman A.B. (2010). Recurrent depressive symptoms and the incidence of dementia and mild cognitive impairment. Neurology.

[10] Borza T., Engedal K., Bergh S., Selbæk G. (2019). Older people with depression—A three-year follow-up. Tidsskr. Nor. Laegeforen..

[11] Oh D.J., Han J.W., Bae J.B., Kim T.H., Kwak K.P., Kim B.J., Kim S.G., Kim J.L., Moon S.W., Park J.H. (2021). Chronic subsyndromal depression and risk of dementia in older adults. Tidsskr. Nor. Legeforen..

[12] Kuring J.K., Mathias J.L., Ward L. (2018). Prevalence of Depression, Anxiety and PTSD in People with Dementia: A Systematic Review and Meta-Analysis. Neuropsychol. Rev..

[13] Park J.H., Lee S.B., Lee T.J., Lee D.Y., Jhoo J.H., Youn J.C., Choo I.H., Choi E.A., Jeong J.W., Choe J.Y. (2007). Depression in vascular dementia is quantitatively and qualitatively different from depression in Alzheimer’s disease. Dement. Geriatr. Cogn. Disord..

[14] Ballard C., Neill D., O’Brien J., McKeith I.G., Ince P., Perry R. (2000). Anxiety, depression and psychosis in vascular dementia: Prevalence and associations. J. Affect. Disord..

[15] Zubenko G.S., Zubenko W.N., McPherson S., Spoor E., Marin D.B., Farlow M.R., Smith G.E., Geda Y.E., Cummings J.L., Petersen R.C. (2003). A collaborative study of the emergence and clinical features of the major depressive syndrome of Alzheimer’s disease. Am. J. Psychiatry.

[16] American Psychiatric Association (2013). Diagnostic and Statistical Manual of Mental Disorders, Fifth Edition: DSM-5.

[17] Burke A.D., Goldfarb D., Bollam P., Khokher S. (2019). Diagnosing and Treating Depression in Patients with Alzheimer’s Disease. Neurol. Ther..

[18] Harrison K.L., Ritchie C.S., Patel K., Hunt L.J., Covinsky K.E., Yaffe K., Smith A.K. (2019). Care Settings and Clinical Characteristics of Older Adults with Moderately Severe Dementia. J. Am. Geriatr. Soc..

[19] Aalten P., Jolles J., de Vugt M.E., Verhey F.R. (2007). The influence of neuropsychological functioning on neuropsychiatric problems in dementia. J. Neuropsychiatry Clin. Neurosci..

[20] Dourado M.C., Laks J. (2016). Psychological interventions for neuropsychiatric disturbances in mild and moderate Alzheimer’s disease: Current evidences and future directions. Curr. Alzheimer Res..

[21] Coyle C.E., Dugan E. (2012). Social isolation, loneliness and health among older adults. J. Aging Health.

[22] Evans I.E.M., Martyr A., Collins R., Brayne C., Clare L. (2018). Social isolation and cognitive function in later life: A systematic review and meta-analysis. J. Alzheimer’s Dis..

[23] Kuiper J.S., Zuidersma M., Oude Voshaar R.C., Zuidema S.U., van den Heuvel E.R., Stolk R.P., Smidt N. (2015). Social relationships and risk of dementia: A systematic review and meta-analysis of longitudinal cohort studies. Ageing Res. Rev..

[24] Alzheimer’s Disease International Overcoming the Stigma of Dementia. https://www.alz.co.uk/sites/default/files/pdfs/world-report-2012-summary-sheet.pdf.

[25] Werner P., Mittleman M.S., Goldstein D., Heinik J. (2012). Family stigma and caregiver burden in Alzheimer’s disease. Gerontologist.

[26] Carers U.K. Carers Manifesto. http://www.carersuk.org/for-professionals/policy/policy-library/carers-manifesto.

[27] Bleijlevens M.H., Stolt M., Stephan A., Zabalegui A., Saks K., Sutcliffe C., Lethin C., Soto M.E., Zwakhalen S.M. (2015). RightTimePlaceCare Consortium. Changes in caregiver burden and health-related quality of life of informal caregivers of older people with Dementia: Evidence from the European RightTimePlaceCare prospective cohort study. J. Adv. Nurs..

[28] Wilks S.E., Croom B. (2008). Perceived stress and resilience in Alzheimer’s disease caregivers: Testing moderation and mediation models of social support. Aging Ment. Health.

[29] Mouton C.P., Haas A., Karmarkar A., Kuo Y.F., Ottenbacher K. (2019). Elder abuse and mistreatment: Results from medicare claims data. J. Elder Abus. Negl..

[30] Max W., Webber P., Fox P. (1995). Alzheimer’s Disease: The Unpaid Burden of Caring. J. Aging Health.

[31] Numbers K., Brodaty H. (2021). The effects of the COVID-19 pandemic on people with dementia. Nat. Rev. Neurol..

[32] Giebel C., Hanna K., Callaghan S., Cannon J., Butchard S., Shenton J., Komuravelli A., Limbert S., Tetlow H., Rogers C. (2021). Navigating the new normal: Accessing community and institutionalised care for dementia during COVID-19. Aging Ment Health.

[33] Canevelli M., Valletta M., Toccaceli Blasi M., Remoli G., Sarti G., Nuti F., Sciancalepore F., Ruberti E., Cesari M., Bruno G. (2020). Facing Dementia During the COVID-19 Outbreak. J. Am. Geriatr. Soc..

[34] Azevedo L.V.D.S., Calandri I.L., Slachevsky A., Graviotto H.G., Vieira M.C.S., Andrade C.B., Rossetti A.P., Generoso A.B., Carmona K.C., Pinto L.A.C. (2021). Impact of Social Isolation on People with Dementia and Their Family Caregivers. J. Alzheimer’s Dis..

[35] Borelli W.V., Augustin M.C., de Oliveira P.B.F., Reggiani L.C., Bandeira-de-Mello R.G., Schumacher-Schuh A.F., Chaves M.L.F., Castilhos R.M. (2021). Neuropsychiatric Symptoms in Patients with Dementia Associated with Increased Psychological Distress in Caregivers During the COVID-19 Pandemic. J. Alzheimer’s Dis..

[36] De Jesús Llibre-Rodríguez J., López A.M., Valhuerdi A., Guerra M., Llibre-Guerra J.J., Sánchez Y.Y., Bosch R., Zayas T., Moreno C. (2014). Frailty, dependency and mortality predictors in a cohort of Cuban older adults, 2003–2011. MEDICC Rev..

[37] World Health Organization (2017). Global Action Plan on the Public Health Response to Dementia 2017–2025.

[38] Alzheimer Europe (2019). Dementia in Europe Yearbook 2019. Estimating the Prevalence of Dementia in Europe.

[39] GBD 2016 Dementia Collaborators (2019). Global, regional, and national burden of Alzheimer’s disease and other dementias, 1990–2016: A systematic analysis for the Global Burden of Disease Study 2016. Lancet Neurol..

[40] Berloto S., Notarnicola E., Perobelli E., Rotolo A. (2020). Italy and the COVID-19 Long-Term Care Situation. Country Report in LTCcovid.org.

[41] Italian Alzheimer’s Disease Association and CENSIS Foundation (Associazione Italiana Malattia di Alzheimer and Fondanzione CENSIS) (2016). L’impatto Economico E Sociale Della Malattia Di Alzheimer: Rifare Il Punto Dopo 16 Anni.

[42] Barbabella F., Poli A., Chiatti C., Pelliccia L., Pesaresi F., NNA Network Non Autosufficienza (2017). The Compass of NNA: The State of the Art Based on Data. Care of Non Self-Sufficient Older People in Italy, 6th Report, 2017–2018.

[43] Tidoli R., NNA Network Non Autosufficienza (2017). Domicialirity. Care of Non Self-Sufficient Older People in Italy. 6th Report, 2017–2018.

[44] Costa G. (2013). Private Assistants in the Italian Care System: Facts and Policies. Obs. Soc. Br..

[45] Alzheimer’s Disease International and Bupa (2013). La Demencia en América: El Coste y la Prevalencia del Alzheimer y Otros Tipos de Demencia.

[46] Gutiérrez-Robledo L.M., Arrieta-Cruz I. (2014). Plan de Acción Alzheimer y Otras Demencias, México 2014.

[47] Gutiérrez-Robledo L.M., Arrieta-Cruz I. (2015). Demencias en México: La necesidad de un Plan de Acción [Dementia in Mexico: The need for a National Alzheimer’s Plan]. Gac. Med. Mex..

[48] Netuveli G., Blane D. (2008). Quality of life in older ages. Br. Med. Bull..

[49] Lam L.C., Tam C.W., Chiu H.F., Lui V.W. (2007). Depression and apathy Affect. functioning in community active subjects with questionable dementia and mild Alzheimer’s disease. Int. J. Geriatr. Psychiatry.

[50] Starkstein S.E., Mizrahi R. (2006). Depression in Alzheimer’s disease. Expert Rev. Neurother..

[51] Tønnesvang J., Sommer U., Hammink J., Sonne M. (2010). Gestalt therapy and cognitive therapy-contrasts or complementarities?. Psychotherapy.

[52] Spagnuolo Lobb M. (2013). The Now-for-Next in Psychotherapy: Gestalt Therapy Recounted in Post-Modern Society.

[53] Spagnuolo Lobb M. (2019). The Paradigm of Reciprocity: How to Radically Respect Spontaneity in Clinical Practice. Gestalt Rev..

[54] Raffagnino R. (2019). Gestalt Therapy Effectiveness: A Systematic Review of Empirical Evidence. Open J. Soc. Sci..

[55] Fuchs T. (2020). Embodiment and personal identity in dementia. Med. Health Care Philos..

[56] Meulmeester F., Francesetti G., Gecele M., Roubal J. (2013). Risk of Psychopathology in Old Age. Gestalt Therapy in Clinical Practice: From Psychopathology to the Aesthetic of Contact.

[57] Spagnuolo Lobb M., Wheeler G. (2015). Fundamentals and development of Gestalt Therapy in the contemporary context. Gestalt Rev..

[58] Spagnuolo Lobb M., Garrety A., Iacono Isidoro S. (2021). Gestalt Perspective on Depressive Experience: A Questionnaire on Some Phenomenological and Aesthetic Dimensions.

[59] Drăghici R. (2011). Experiential Psychotherapy in Geriatric Groups. Procedia Soc. Behav. Sci..

[60] Azermai M., Petrovic M., Elseviers M.M., Bourgeois J., Van Bortel L.M., Vander Stichele R.H. (2012). Systematic appraisal of dementia guidelines for the management of behavioural and psychological symptoms. Ageing Res. Rev..

[61] de Oliveira A.M., Radanovic M., de Mello P.C., Buchain P.C., Vizzotto A.D., Celestino D.L., Stella F., Piersol C.V., Forlenza O.V. (2015). Nonpharmacological Interventions to Reduce Behavioral and Psychological Symptoms of Dementia: A Systematic Review. Biomed Res. Int..

[62] Leyhe T., Reynolds C.F., Melcher T., Linnemann C., Klöppel S., Blennow K., Zetterberg H., Dubois B., Lista S., Hampel H. (2017). A common challenge in older adults: Classification, overlap, and therapy of depression and dementia. Alzheimer’s. Dement..

[63] Moraros J., Nwankwo C., Patten S.B., Mousseau D.D. (2017). The association of antidepressant drug usage with cognitive impairment or dementia, including Alzheimer disease: A systematic review and meta-analysis. Depress Anxiety..

[64] Leong C. (2014). Antidepressants for depression in patients with dementia: A review of the literature. Consult Pharm..

[65] Orgeta V., Qazi A., Spector A., Orrell M. (2015). Psychological treatments for depression and anxiety in dementia and mild cognitive impairment: Systematic review and meta-analysis. Br. J. Psychiatry.

[66] Cheston R., Ivanecka A. (2017). Individual and group psychotherapy with people diagnosed with dementia: A systematic review of the literature. Int. J. Geriatr. Psychiatry.

[67] American Psychological Association (2006). Evidence-based practice in psychology. Am. Psychol..

[68] Krasny-Pacini A., Evans J. (2018). Single-case experimental designs to assess intervention effectiveness in rehabilitation: A practical guide. Ann. Phys. Rehabil. Med..

[69] Regulation (EU) 2016/679 of the European Parliament and of the Council of 27 April 2016 on the Protection of Natural Persons with Regard to the Processing of Personal Data and on the Free Movement of Such Data, and Repealing Directive 95/46/EC (General Data Protection Regulation). https://www.legislation.gov.uk/eur/2016/679/contents.

[70] Herrera P., Brownell P., Roubal J., Mstibovskyi I., Glänzer O., La Rosa R., Tosi S. (2020). Progetto Gestaltico di Collaborazione Internazionale: Il Caso Singolo, Progetto di Ricerca con Serie Temporali. Manuale per Ricercatori.

[71] Burke W.J., Miller J.P., Rubin E.H., Morris J.C., Coben L.A., Duchek J., Wittels I.G., Berg L. (1988). Reliability of the Washington University Clinical Dementia Rating. Arch. Neurol..

[72] Kørner A., Lauritzen L., Abelskov K., Gulmann N., Marie Brodersen A., Wedervang-Jensen T., Marie Kjeldgaard K. (2006). The Geriatric Depression Scale and the Cornell Scale for Depression in Dementia. A validity study. Nord. J. Psychiatry.

[73] Cummings J.L., Mega M., Gray K., Rosenberg-Thompson S., Carusi D.A., Gornbein J. (1994). The Neuropsychiatric Inventory: Comprehensive assessment of psychopathology in dementia. Neurology.

[74] Binetti G., Mega M.S., Magni E., Padovani A., Rozzini L., Bianchetti A., Cummings J., Trabucchi M. (1998). Behavioral disorders in Alzheimer’s Disease: A transcultural perspective. Arch. Neurol..

[75] Zepeda M.U.P., Guerrero J.A.R., Carrasco O.R., Robledo L.M.G. (2008). P3-038: Validation of the neuropsychiatric inventory questionnaire in a group of Mexican patients with dementia. Alzheimer’s Dement..

[76] Kaufer D.I., Cummings J.L., Christine D., Bray T., Castellon S., Masterman D., MacMillan A., Ketchel P., DeKosky S.T. (1998). Assessing the impact of neuropsychiatric symptoms in Alzheimer’s disease: The Neuropsychiatric Inventory Caregiver Distress Scale. J. Am. Geriatr. Soc..

[77] Hughes C.P., Berg L., Danziger W.L., Coben L.A., Martin R.L. (1982). A new clinical scale for the staging of dementia. Br. J. Psychiatry.

[78] Morris J.C. (1993). The Clinical Dementia Rating (CDR): Current version and scoring rules. Neurology.

[79] O’Bryant S.E., Waring S.C., Cullum C.M., Hall J., Lacritz L., Massman P.J., Lupo P.J., Reisch J.S., Doody R. (2008). Texas Alzheimer’s Research Consortium Staging Dementia Using Clinical Dementia Rating Scale Sum of Boxes Scores: A Texas Alzheimer’s Research Consortium Study. Arch. Neurol..

[80] Yeo C.Y.Y., Chan M.P.C., Lim W.S., Chong M.S. (2010). P2-100: Clinical Utility of the Clinical Dementia Rating Sum of Boxes in Mild Cognitive Impairment and Dementia in an Asian Population. Alzheimer’s Dement..

[81] Battle C.C., Imber S.D., Hoehn-Saric R., Nash E.R., Frank J.D. (1966). Target complaints as criteria of improvement. Am. J. Psychother..

[82] Shorer C. (1970). Improvement with and without psychotherapy. Dis. Nerv. Syst..

[83] Frey J., Heckel R.V., Salzberg H.C., Wackwitz J. (1976). Demographic variables as predictors of outcome in psychotherapy with children. J. Clin. Psychol..

[84] Russell D.W. (1996). UCLA Loneliness Scale (Version 3): Reliability, validity, and factor structure. J. Pers. Assess..

[85] Boffo M., Mannarini S., Munari C. (2012). Exploratory Structure Equation Modeling of the UCLA Loneliness Scale: A contribution to the Italian adaptation. TPM.

[86] Sancho P., Pinazo-Hernandis S., Donio-Bellegarde M., Tomás J.M. (2020). Validation of the University of California, Los Angeles Loneliness Scale (version 3) in Spanish older population: An application of exploratory structural equation modelling. Aust. Psychol..

[87] Logsdon R.G., Gibbons L.E., McCurry S.M., Teri L. (1999). Quality of life in Alzheimer’s disease: Patient and caregiver reports. J. Ment. Health Aging.

[88] Zarit S.H., Reever K.E., Back-Peterson J. (1980). Relatives of the impaired elderly: Correlates of feelings of burden. Gerontologist.

[89] Hérbert R., Bravo G., Préville M. (2000). Reliability, validity, and reference values of the Zarit Burden Interview for assessing informal caregivers of community-dwelling older persons with dementia. Can. J. Aging.

[90] Zarit Burden Interview (ZBI). https://eprovide.mapi-trust.org/instruments/zarit-burden-interview.

[91] Archbold P.G., Stewart B.J., Greenlick M.R., Harvath T. (1990). Mutuality and preparedness as predictors of caregiver role strain. Res. Nurs. Health.

[92] Godwin K.M., Swank P.R., Vaeth P., Ostwald S.K. (2013). The longitudinal and dyadic effects of mutuality on perceived stress for stroke survivors and their spousal caregivers. Aging Ment. Health.

[93] Lyons K.S., Stewart B.J., Archbold P.G., Carter J.H. (2009). Optimism, pessimism, mutuality, and gender: Predicting 10-year role strain in Parkinson’s disease spouses. Gerontologist.

[94] Halm M.A., Treat-Jacobson D., Lindquist R., Savik K. (2007). Caregiver burden and outcomes of caregiving of spouses of patients who undergo coronary artery bypass graft surgery. Heart Lung.

[95] Froyd J.E., Lambert M.J., Froyd J.D. (1996). A review of practices of psychotherapy outcome measurement. J. Ment. Health.

[96] Evans C., Connell J., Barkham M., Margison F., McGrath G., Mellor-Clark J., Audin K. (2002). Towards a standardised brief outcome measure: Psychometric properties and utility of the CORE–OM. Br. J. Psychiatry.

[97] Barkham M., Culverwell A., Spindler K., Twigg E. (2005). The CORE-OM in an older adult population: Psychometric status, acceptability, and feasibility. Aging Ment. Health.

[98] Fogarty M., Bhar S., Theiler S. (2020). Development and validation of the Gestalt Therapy Fidelity Scale. Psychother. Res..

[99] Borckardt J.J., Nash M.R., Murphy M.D., Moore M., Shaw D., O’Neil P. (2008). Clinical practice as natural laboratory for psychotherapy research: A guide to case-based time-series analysis. Am. Psychol..

[100] Campbell J.M. (2003). Efficacy of behavioral interventions for reducing problem behavior in persons with autism: A quantitative synthesis of single-subject research. Res. Dev. Disabil..

[101] Olive M.L., Smith B.W. (2005). Effect size calculations and single subject designs. Educ. Psychol..

[102] Delfs C.H., Campbell J.M. (2010). A quantitative synthesis of developmental disability research: The impact of functional assessment methodology on treatment effectiveness. Behav. Anal. Today.

[103] Kitwood T. (1997). The experience of dementia. Aging Ment. Health.

[104] Dewing J. (2008). Personhood and dementia: Revisiting Tom Kitwood’s ideas. Int. J. Older People Nurs..

[105] Herrera P., Mstibovskyi I., Roubal J., Brownell P. (2020). The Single-Case, Time-Series Study. Int. J. Psychother..

[106] Aminzadeh F., Byszewski A., Molnar F.J., Eisner M. (2007). Emotional impact of dementia diagnosis: Exploring persons with dementia and caregivers’ perspectives. Aging Ment. Health.

[107] Bopp-Kistler I. (2015). Diagnoseeröffnung und Begleitung [Disclosing the diagnosis and guidance]. Ther. Umsch..

